# Erratum

**DOI:** 10.5423/PPJ.ER.08.2015.0173

**Published:** 2016-04-01

Minji Kang^1,†^, Jang Kyun Seo^2,†^, Hoseong Choi^1^, Hong Soo Choi^2^ and Kook Hyung Kim^1,3,^*. Establishment of a Simple and Rapid Gene Delivery System for Cucurbits by Using Engineered of *Zucchini yellow mosaic virus*. *Plant Pathol. J.* 32(1):70–76 (2016).

Title contained an error.

Establishment of a Simple and Rapid Gene Delivery System for Cucurbits by Using Engineered of *Zucchini yellow mosaic virus*

The corrected title follows below (bold in text).

Establishment of a Simple and Rapid Gene Delivery System for Cucurbits by Using Engineered **Zucchini Yellow Mosaic Virus**

